# Comparison of the composition, immunological activity and anti-fatigue effects of different parts in sika deer antler

**DOI:** 10.3389/fphar.2024.1468237

**Published:** 2024-12-19

**Authors:** Siqi Chen, Yidan Li, Yichun Yang, Shibo Zhao, Huali Shi, Chengkai Yang, Min Wu, Aiwu Zhang

**Affiliations:** College of Animal Science and Technology, Jilin Agricultural University, Changchun, China

**Keywords:** sika deer antler, composition, immunological activity, anti-fatigue effect, traditional medicine

## Abstract

**Background:**

Sika deer (*Cervus nippon Temminck*, 1838) antler is a highly esteemed tonic renowned for its abundant assortment of polypeptides, polysaccharides, amino acids, and minerals, and is recognized for its multifarious pharmacological properties. However, limited research has been conducted regarding the variation in composition of deer antlers between the upper and basal sections, as well as their pharmacological effects on immunological activity and anti-fatigue in mice. The objective of this study was to conduct a comprehensive analysis on the appearance, chemical composition, and pharmacological effects of different components within sika deer antlers. This investigation aims to elucidate the disparities in quality among various parts of antlers and establish a theoretical foundation for the precise utilization of sika deer antlers.

**Methods:**

The contents of protein, amino acids, polysaccharides, phospholipids, minerals and nucleotides in wax, powder, gauze and bone slices were determined by different nutrient assays. Then, 100 mice were randomly divided into 5 groups. The mice in control group were administered 0.3 mL of saline solution per day. The mice in experimental groups were administered 0.3 mL enzymatic hydrolysate of the wax slice, powder slice, gauze slice, bone slice separately per day, continuously for 14 days from the first day. The effect of antler on boosting immunity was evaluated by testing organ indices and assessing immunoglobulin levels by ELISA. Anti-fatigue effects were assessed by a mouse swimming test. Finally, the correlation between composition and pharmacological effects was analysed.

**Results:**

The content of each marker substance gradually decreases from the upper to the basal of deer antler. The protein and uracil content in the wax slice were significantly higher than the other three groups (*p* < 0.05), and the phospholipid and inosine content were strongly significantly higher than the other three groups (*p* < 0.01). The content of polysaccharides and hypoxanthine in the wax slice group and powder slice group was significantly higher than that in the gauze slice group and bone slice group (*p* < 0.05). The amino acid content decreases from the upper to the basal section. Among, the content of Glu, Gly, His, and Pro wax slice was significantly higher than the other three groups (*p* < 0.01). The content of other minerals except Fe and Mg in the wax slice group was significantly higher than the other three groups (*p* < 0.01), and the content of Fe and Mg in the bone slice was the highest. Additionally, the immune organ index, immunoglobulin, and glycogen contents displayed a significant increase in comparison to both the control group and the other experimental groups (*p* < 0.05). And the swimming endurance of mice in the wax slice group was significantly prolonged (*p* < 0.01). The skeletal muscle state of the wax group mice exhibited superior characteristics, characterized by distinct horizontal stripes and tightly arranged muscle fibers. In contrast, the bone group displayed noticeable yet relatively less compact horizontal stripes. Among the organic and inorganic compositions of deer antler, the highest degree of correlation with the content of IgA, IgM, and IgG was found to be protein (r^2^ = 0.999), uracil (r^2^ = 0.987), and inosine (r^2^ = 0.999), respectively. The proteins (r^2^ = 0.997) appear to exert a significant influence on the anti-fatigue effect, while polysaccharides (r^2^ = 0.865) demonstrate the least relevance.

**Conclusion:**

These outcomes indicated that the wax slice yielded optimal results among the tested parts and demonstrated the highest efficacy.

## 1 Introduction

Deer antler refers to the immature horn of male deer, a member of the Cervidae family, characterized by densely packed antler hair that has not yet undergone ossification. The investigation of deer antler has long been a prominent subject in the realm of traditional pharmacological research, with prior studies demonstrating its diverse array of inorganic and organic compounds. The former primarily consists of various micronutrients (Dong et al., 2004), whereas the latter predominantly encompasses polysaccharides, phospholipids, nucleosides, lipid-soluble and water-soluble compounds, as well as insoluble proteins. These chemical constituents are intricately associated with the pharmacological properties of deer antler (Liu et al., 2020).

Two millennia ago, ancient medical literature provided a comprehensive account of the therapeutic application of sika deer antlers (*Cervus nippon Temminck*, 1838) for treating a diverse range of 52 ailments (Kawtikwar et al., 2010). In recent years, the application of genomics, proteomics, and other molecular biology techniques has significantly broadened the scope of research on marker substances and medicinal mechanisms associated with deer antler. However, despite the continuous expansion of this scope, there remains a limited focus on evaluating the efficacy of different components within deer antler. The minimal dosage, rapid therapeutic effects, and positive health outcomes of Sika deer antler and its derivatives have garnered significant attention from researchers both domestically and internationally. In terms of immune enhancement, Zheng (Zheng et al., 2017) demonstrated that oral administration of deer antler in mice resulted in an increased proportion of T cells and a decreased proportion of B cells in peripheral blood. Scientific evidence has demonstrated the significant impact of deer antler on augmenting cellular immunity within the human body.

Meanwhile, Liu and college (Liu et al., 2023) elucidated the potential regulatory mechanism of deer antler in enhancing immunity through network pharmacology and molecular docking technology, and identified four substances and 130 core targets that may exert immune regulatory effects. Deer antler extract also exerts a significant impact on the process of fracture healing. The findings reported by Wang (Wang et al., 2022) demonstrate that deer antler extract effectively stimulates fracture healing through the activation of the BMP-2/SMAD4 signaling pathway. Deer antler also exhibits a notable anti-fatigue effect. The findings of Zhang (Zhang et al., 2020) demonstrated a direct correlation between the levels of eight nucleotides in deer antler, spanning from the upper to basal section, and the forced swimming test performance in mice. Therefore, it can be inferred that deer antler exhibits a significant anti-fatigue effect. Undoubtedly, deer antler exhibits potent pharmacological properties with anti-inflammatory effects. Widyowati (Widyowati et al., 2023) established a mouse model of arthritis and demonstrated the efficacy of orally administered ethanol extract of deer antler in reducing IL-1β cytokine levels. The present study aims to demonstrate the efficacy of deer antler in reducing the levels of inflammatory factors, thereby highlighting its significant potential in combating inflammation. The diverse chemical composition of deer antler serves as the foundation for its multifaceted medicinal properties. The intricate chemical composition of deer antler underscores the intricacy of its pharmacological potential. Furthermore, the intricate interplay between deer antler quality, marker substances, and concentration underscores the multifaceted relationship between the quality of deer antler and its pharmacological effects.

**FIGURE 1:**
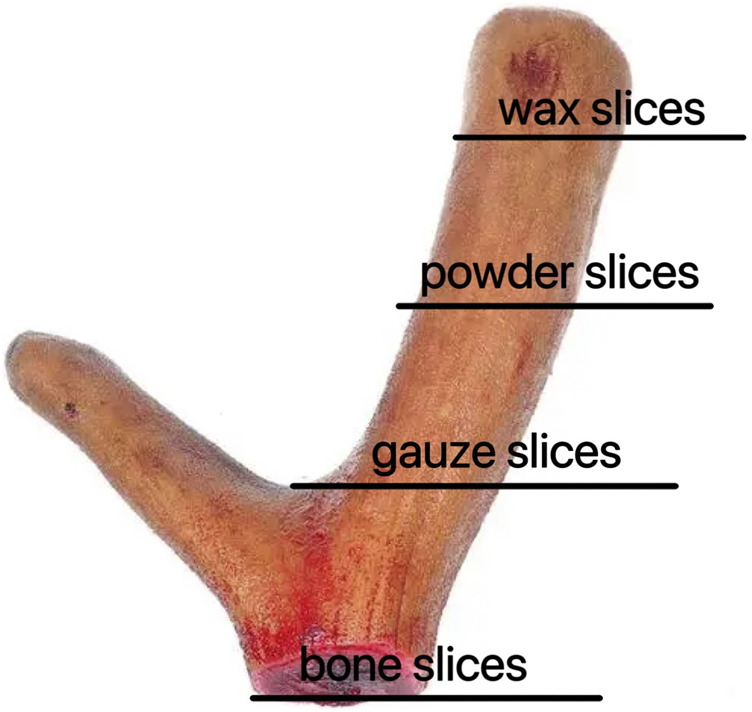
Different parts of deer antler in sika deer (from the upper to the basal section).

To date, numerous studies have been conducted to investigate the comprehensive pharmacological effects of deer antler. However, limited research has been conducted on the fractionation of deer antler to investigate its chemical composition and pharmacological variations. Hence, the objective of this study is to conduct a comparative analysis on the composition of four distinct segments of deer antlers, namely, wax slice, powder slice, gauze slice, and bone slice (Figure 1). Furthermore, we endeavored to assess the immunomodulatory activity and anti-fatigue efficacy of deer antler hydrolysate in mice. Through this study, our objective is to provide valuable insights into the pharmacological effects and therapeutic mechanisms of deer antler, facilitating a comprehensive exploration in line with the rigorous standards set by Nature journal.

## 2 Materials and methods

### 2.1 Sample collection

Sika deer antlers in the early stages of growth and development were provided by the Dongda Deer Farm in Changchun, Jilin Province, China. The deer was first placed under general anesthesia and the antler was then removed proximally using a surgical hand saw (Shi et al., 2021). The studies were approved by the Experimental Animal Welfare and Ethics Committee of Dongda Deer Farm (permit number: 20230810001). The studies were conducted in accordance with the local legislation and institutional requirements.

### 2.2 Reagents and antibodies

Copper sulfate (CAS: 7758-98-7), potassium sulfate (CAS: 7778-80-5), sulfuric acid (CAS: 7664-93-9), 2% boric acid solution, nitric acid (CAS: 7697-37-2), methyl red bromocresol green mixed indicator, 40% sodium hydroxide solution (CAS: 1310-73-2), boric acid (CAS: 10043-35-3), ethanol (CAS: 64-17-5), phenol (CAS: 108-95-2), high-purity nitrogen gas, sodium citrate (CAS: 68-04-2), elution buffer solution, indanone solution (CAS: 485-47-2), amino acid standard, Sodium molybdate (CAS: 7631-95-0), potassium dihydrogen phosphate (CAS: 7778-77-0), 50% potassium hydroxide solution (CAS: 1310-58-3), hydrochloric acid (CAS: 7647-01-0), zinc oxide (CAS: 1314-13-2), 0.015% hydrazine sulfate solution (CAS: 10034-93-2), analytical pure glucose, trichloroacetic acid (CAS: 76-03-9) Potassium borohydride (CAS: 13762-51), ammonium molybdate (CAS: 13106-76-8), hydroquinone (CAS: 123-31-9), all reagents are analytical grade and come from Beijing Chemical Plant; Uracil (batch number TM0313XB13), hypoxanthine (batch number TM0313XC13), inosine (batch number TJ0623XA13) were procured from Shanghai Yuanye Biotechnology Co., Ltd. (Shanghai, China).; The mass concentration of the multi-element mixed standard reserve solution of Zn and Fe, as well as the single element standard reserve solution of K, Na, and Mg, are both 1,000 μg·L^−1^, provided by the National Nonferrous Metals and Electronic Materials Analysis and Testing Center. Pepsin: 1:3,000, purchased from Changchun Huayi Biotechnology Co., Ltd. Paraformaldehyde (4%), hematoxylin and eosin were supplied by Biosharp, Hefei, China.

### 2.3 Appearance of different parts of sika deer antlers slice

Six two-branched fresh deer antlers were cut from the upper to the basal section into four separate parts: wax slice, powder slice, gauze slice and bone slice. The fresh two-branched sika deer antlers were meticulously examined to assess variations in hue, texture, and fragrance following precise slicing using a specialized slicer, facilitating a comprehensive comparison across different sections of the antlers.

### 2.4 Sika deer antler powder and its enzymatic hydrolysate preparation

The wax slice, powder slice, gauze slice and bone slice of each deer antler were ground and mixed together separately to pass through a 250 mm screen and stored for subsequent analysis. Twelve grams of different parts of deer antler powder were weighed and dissolved in 50 mL of distilled water. Subsequently, a 1% hydrochloric acid solution was added, and the pH was adjusted to between 1.5 and 2. Following this, 0.24 g of pepsin was added in accordance with a 1:50 ratio of enzyme to substrate to initiate the enzymatic hydrolysis. The enzymatic digestion process was consistently maintained in a 37°C constant temperature water bath. After 6 h of enzymatic digestion, a 1% sodium hydroxide solution was introduced to adjust the pH of the enzymatic digestion solution to 6.5–7.0. Once the enzymatic digestion was complete, the solution was subjected to boiling for 10 min, followed by cooling to room temperature. Subsequently, centrifugation was carried out for 15 min at 3,500 rpm, and the resulting supernatant was collected and stored at 4°C for future use.

### 2.5 Chemical composition analysis of different parts in sika deer antlers

#### 2.5.1 Determination of protein content

The protein was determined using the Bradford method and the absorbance was measured using a UV spectrophotometer. Make a suspension of deer antler powder and set it aside. Extract water-soluble protein using a 200W ultrasonic cleaner, centrifuge the supernatant after ultrasonic treatment at a speed of 4000r/min. Prepare a standard solution using bovine serum albumin (BSA) and create a standard curve. Add the extracted supernatant to Coomassie Brilliant Blue-250, mix well, and measure the absorbance after 10 min. Based on the absorbance of the sample to be tested, calculate the protein concentration of the sample from the standard curve.

#### 2.5.2 Evaluation of phospholipids content

Phospholipids were measured according to the method of Nation Standards of the People’s Republic of China GB/T 5537-2008. A precise 10 g of different parts of deer antler powder was meticulously weighed and subjected to ashing with zinc oxide. Subsequently, the resulting ash was dissolved in 10 mL of hot hydrochloric acid (1:1) and meticulously filtered. The filtrate was then neutralized with a 50% potassium hydroxide solution until turbidity appeared. Following this, hydrochloric acid was cautiously added dropwise to ensure the dissolution of all zinc hydroxide precipitates. Two drops were added, and the sample was ultimately diluted with water to the specified scale and thoroughly shaken. The phospholipid content was then determined using a UV spectrophotometer (SPECORD 205, Germany).

#### 2.5.3 Assessment of polysaccharides content

Polysaccharides were measured according to the method of Nation Standards of the People’s Republic of China GB/T 40632-2021. Weigh 1.000 g of deer antler powder, add 1 mL of a 15% trichloroacetic acid solution and a small amount of a 5% trichloroacetic acid solution, and mix thoroughly. Centrifuge three times at 3,000 rpm, collecting the supernatant each time. Add 2 mL of 6 mol/L hydrochloric acid to the supernatant, mix well, and then place in a 96°C water bath for 2 h. After the water bath, add 2 mL of 6 mol/L sodium hydroxide solution to a 25 mL volumetric flask, then add 6% phenol solution and concentrated sulfuric acid, and mix them well. Prepare the test solution, measure the absorbance at 490 nm using a UV spectrophotometer, and calculate the polysaccharide content based on the standard curve.

#### 2.5.4 Evaluation of nucleosides content

The nucleosides were measured according to the method established by Sun et al. (2018). A precise 100 mg of different parts of deer antler powder was extracted using ultrasonic extraction with distilled water, and the resulting supernatant was filtered through a 0.22 μm aqueous filtration membrane. The content of nucleosides was determined using the UPLC method.

#### 2.5.5 Determination of amino acid content

Amino acid were measured according to the method of Nation Standards of the People’s Republic of China GB 5009.124–2016. For the analysis of amino acids, 30 mg of samples was accurately weighed following the hydrolysis of different parts of deer antler powder. The resulting samples were then meticulously filtered using a syringe filter (0.22 μm) and subjected to analysis using an amino acid autoanalyzer (Pharmacia Biotech Biochrom 20, Ninhydrin Method). The determination of amino acids was conducted based on absorbances recorded at 440 and 570 nm.

#### 2.5.6 Assessment of mineral content

The determination of mineral content was conducted following the method outlined by Wang et al. (2018). The concentrations of potassium (K), sodium (Na), and iron (Fe) were analyzed using atomic absorption spectrometry, while magnesium (Mg) and zinc (Zn) were determined using ICP-MS.

### 2.6 Animal experiment

#### 2.6.1 Animals

One hundred mice used in the experiment were procured from Liaoning Changsheng Biotechnology Co., Ltd. (Changchun, China) and were identified with license number SCXK (Liaoning) 2020-0001, as well as an experimental animal quality certificate number: NO.210726230101468567. Throughout the experiment, the mice were maintained in an environment with a constant temperature of 23°C, adequate humidity, access to abundant water and food, and 12-h light-dark cycle. The experimental protocol, which was meticulously crafted to alleviate pain and discomfort for the animals, received approval from the Animal Experiment Ethics Committee of Jilin Agricultural University (permit number: 20240703001). Mice were individually housed and underwent a 12-h fasting period prior to the experiments. All co-authors were in complete agreement with the plans and procedures of the animal experiments.

#### 2.6.2 Experimental design

One hundred 8-week-old KM male mice were adaptively raised for 1 week, and then randomly divided into 5 groups (n = 20) including 1 control group and 4 experimental groups. The mice in control group were administered 0.3 mL of saline solution per day. The mice in experimental groups were administered 0.3 mL enzymatic hydrolysate of the wax slice, powder slice, gauze slice, bone slice separately per day, continuously for 14 days from the first day. 10 mice were used to compare the effects of different parts of deer antler on immunological activity in mice, and other 10 mice were used to compare the effects of different parts of deer antler on anti-fatigue in mice. Fourteen days later, blood samples were procured from the ocular region, and the mice were euthanized through cervical dislocation. Subsequently, tissue samples including the spleen, thymus, and muscle were harvested from the deceased mice (Lu Y et al., 2024). Serum was obtained after the collected blood sample was centrifuged at 3,500 rpm at 4°C for 10 min and stored at −20°C for subsequent immunoglobulins analysis experiments.

#### 2.6.3 Immune organ index analysis

The overall health of the mice, including their appearance, activity, and feeding behavior, was meticulously monitored throughout the experiment. Following the treatment period, the mice were humanely sacrificed, and their spleens and thymuses were promptly excised and weighed to determine the thymus index and spleen index. The index were calculated using the following formula:

$$\text{Index}\left(\text{mg}/g\right)=\frac{\text{weight}\;\text{of}\;\text{thymus}\;\text{or}\;\text{spleen}}{\text{body}\;\text{weight}}$$

#### 2.6.4 Immunoglobulins analysis

The levels of immunoglobulin A (IgA), immunoglobulin G (IgG), and immunoglobulin M (IgM) in the blood were quantified using commercially available kits obtained from Nanjing Jiancheng Institute of Biotechnology (Nanjing, China) in accordance with the manufacturer’s instructions.

#### 2.6.5 Anti-fatigue analysis

After the completion of the intragastric administration regimen, on the 15th day, lead blocks weighing 8% of the mice’s body weight were affixed to their tails. Subsequently, the mice were subjected to a forced swimming test in a water tank while carrying additional weight load. The swimming pool had a depth of approximately 35 cm, while the water temperature was maintained at (25 ± 2)°C throughout the study period. The duration of swimming under the imposed weight, from the initiation of swimming until the mice submerged and succumbed, was recorded to assess the impact of anti-fatigue.

#### 2.6.6 Glycogen levels analysis

##### 2.6.6.1 Sample preparation

After the completion of the intragastric administration regimen on the 15th day, mice were euthanized and their livers were excised. The skeletal muscles from the thighs of the mice were meticulously dissected in a circular fashion using a scalpel. Subsequently, 0.2 g of liver/muscle tissue was accurately weighed and transferred into a 10 mL centrifuge tube for subsequent homogenization. A 1 mL aliquot of the resulting homogenate was subsequently subjected to centrifugation at a speed of 8000 × g for a duration of 10 min at room temperature, and the resultant supernatant was collected as the test sample.

The reagents listed in Table 1 were then added sequentially to the centrifuge tube.

**TABLE 1:** Reagents to be added to each tube.

Reagents	Measuring tube (μL)	Standard tube (μL)	Empty tube (μL)
Sample	60	—	—
Standard liquid	—	60	—
Distilled water	—	—	60
Colour liquid	240	240	240

Boiling water bath for 10 min (sealed to prevent water loss) Cool to room temperature.

##### 2.6.6.2 Measure of absorbances

Pipette 200 μL of the reaction solution into a 96-well plate or microglass cuvette and measure the absorbance value at 620 nm, which is recorded as the A assay, A standard and A blank; calculate ∆A assay = A assay-A blank and ∆A standard = A standard-A blank.

##### 2.6.6.3 Establishment of standard curve

The standard curve was plotted with 0.30, 0.25, 0.20, 0.15, 0.10, 0.05 mg/mL as the horizontal coordinate (x) and its corresponding ΔA standard as the vertical coordinate (y) to obtain the standard equation y = kx + b, and the ΔA measurement was brought into the formula to obtain x (mg/mL).

##### 2.6.6.4 Formula for calculating glycogen

Glycogen mg/g=x×V×sample total0.9 / W=4.5×x /W.

(V _sample total_: total volume of sample to be measured, 5 mL; W: sample mass, g).

#### 2.6.7 Pathological and histological examination

To prepare the histological slices, samples of leg muscle were first fixed in 4% paraformaldehyde, then embedded in paraffin and subsequently sectioned. The resulting tissue slices were stained with hematoxylin and eosin (H&E) from Biosharp, China, and captured under anoil microscope (Nikon Eclipse 80i, Tokyo, Japan).

### 2.7 Generation of correlation heat maps

The correlation heat map was generated by the Lianchuan BioCloud platform, with the Pearson method selected for the correlation analysis. The color gradient ranging from blue to red signifies the continuum of correlation, spanning from negative to positive values, while the numerical representation on the graph denotes the r^2^ (correlation coefficient).

### 2.8 Statistical analysis

Statistical analysis was conducted using least-squares analysis of variance (ANOVA) in accordance with the general linear model procedure of SPSS (SPSS 26.0 for Windows; SPSS Inc., Chicago, IL, United States). Each experiment was replicated at least three times. Descriptive statistics were presented as mean ± standard deviation ( $\bar{x}$ ± SD), and comparisons were made using one-way ANOVA followed by the Duncan test. A significance level of *p* < 0.01 was considered highly significant, while *p* < 0.05 was deemed significant.

## 3 Results

### 3.1 Assessment of visual characteristics

**FIGURE 2:**
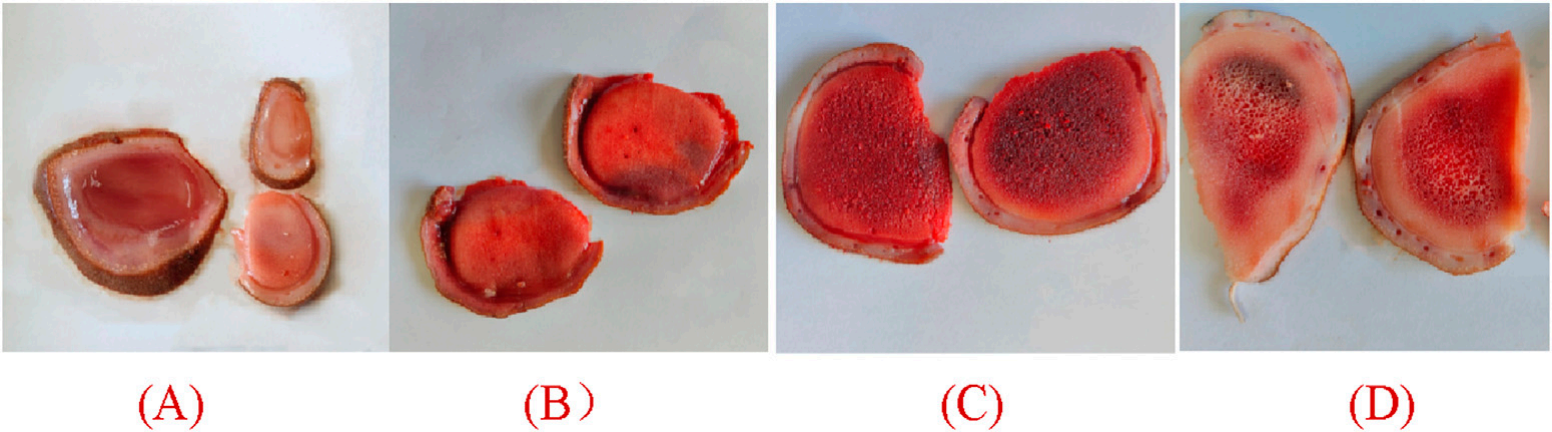
Appearance of different parts of deer antlers slice [In the panel **(A)** wax slice, **(B)** powder slice, **(C)** gauze slice, **(D)** bone slice].

Wax slice: The slices are cut into circular or elliptical shapes. They display a light reddish-brown hue, translucency, a subtle gloss, and a reddish-brown rim along the periphery. They lack osseous structures and exhibit a resilient, compact, lipid-rich, and moist constitution reminiscent of adipose tissue. The color of the substance is a waxy yellow, exhibiting a crystalline appearance reminiscent of beeswax, which signifies its exceptional quality (Figure 2A).

Powder slice: The slices are uniformly round and slightly thick, exhibiting a neat surface texture. The specimens exhibit a smooth texture, devoid of any bony structures, and are characterized by a distinctive yellow-brown peripheral ring. Recurring instances of residual deer antler may occasionally be observed. The fishy flavor is subtly present, accompanied by a subtle hint of salinity, suggesting a lower quality in comparison to wax slices (Figure 2B).

Gauze slice: The slices are circular and substantial, showcasing a meticulous surface adorned with densely distributed honeycomb perforations and delicate filamentous apertures. The specimens demonstrate no yarn leakage and exhibit a seamless integration between the cortex and deer antler tissues. Possessing a slightly granular texture and exhibiting a yellow-brown ring at the periphery, they are comparatively coarser than powdered slices, indicating inferior quality (Figure 2C).

Bone slice: The slice exhibits a white, honeycomb-like structure with substantial interstices. The outer layer exhibits an osseous structure, contributing to a robust and brittle characteristic, rendering them susceptible to fracturing (Figure 2D).

### 3.2 Determination of protein, phospholipid, and polysaccharide levels

**FIGURE 3:**
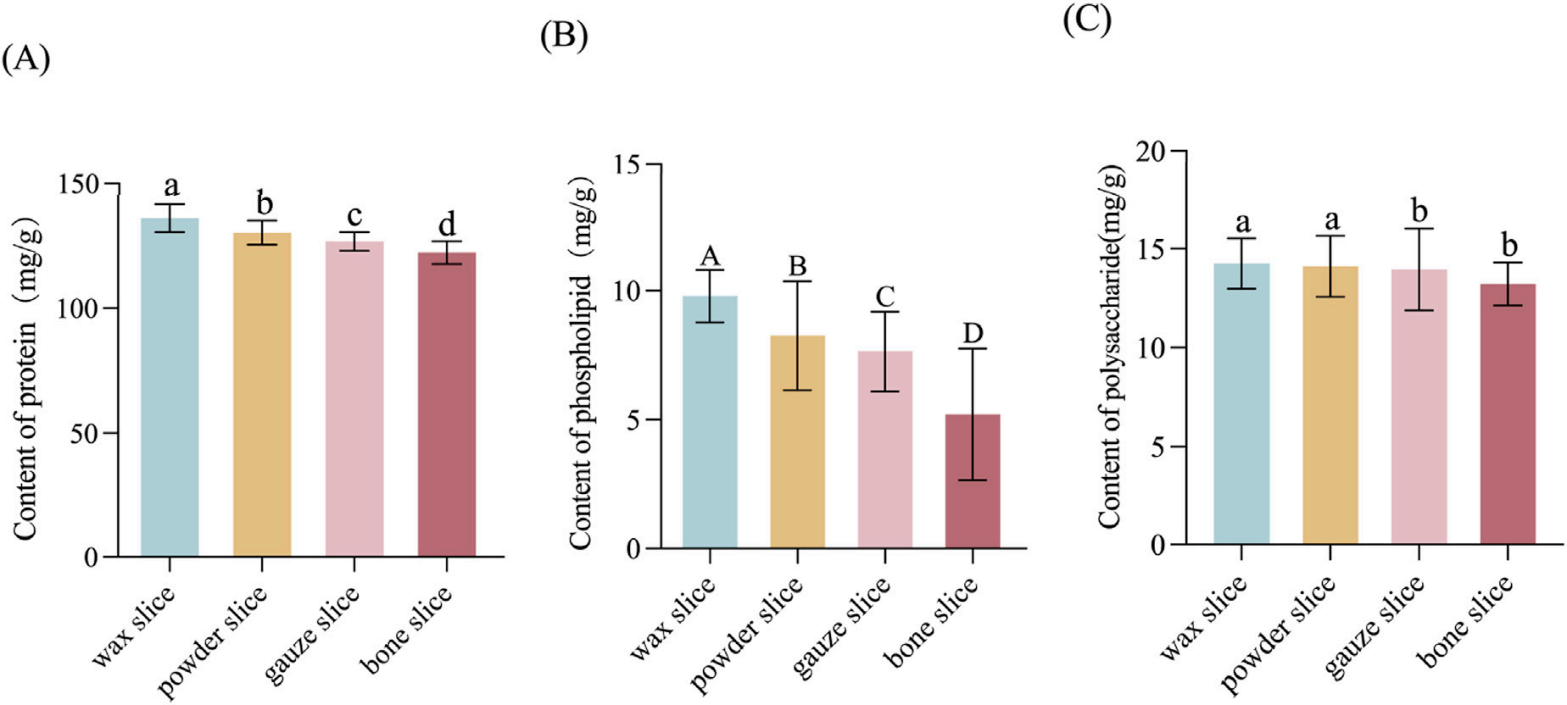
The protein, phospholipid and polysaccharide contents in different parts of deer antler **(A)** Content of protein in different parts of deer antler. **(B)** Content of phospholipid in different parts of deer antler. **(C)** Content of polysaccharide in different parts of deer antler. Note: Means for different lowercase letters were significantly different (*p* < 0.05). Means for different capital letters were extremely significantly different (*p* < 0.01).

The data indicate a sequential decrease in protein content from wax slice to bone slice, with a significant variance among the four parts (*p* < 0.05, Figure 3A). Similarly, the phospholipid content exhibited a sequential reduction from wax slice to bone slice, with a highly significantly variance among the four parts (*p* < 0.01, Figure 3B). Furthermore, the polysaccharide content was notably higher in wax slice and powder slice compared to gauze slice and bone slice, the difference was significant (*p* < 0.05, Figure 3C).

### 3.3 Determination of nucleosides contents

**FIGURE 4:**
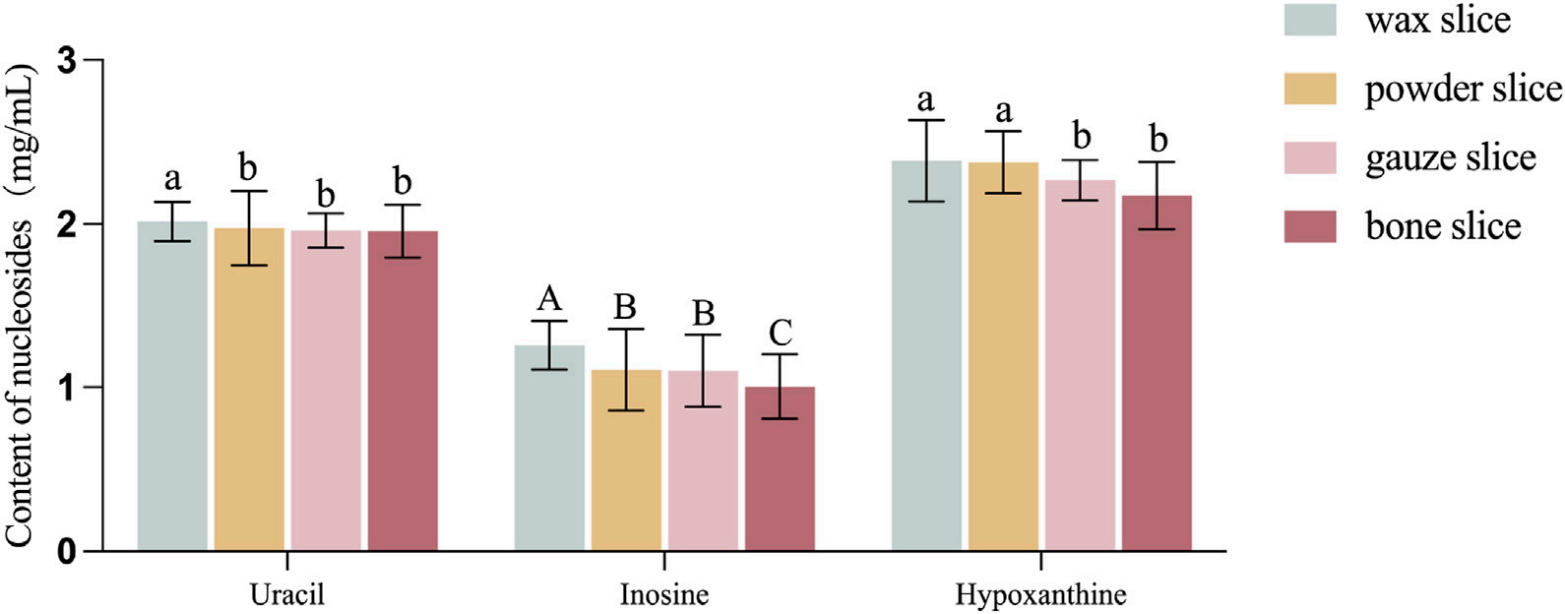
Content of nucleosides in different parts of deer antler. Note: Means for different lowercase letters were significantly different (*p* < 0.05). Means for different capital letters were extremely significantly different (*p* < 0.01).

The Figure 4 illustrates the levels of uracil, inosine and hypoxanthine in various parts of deer antlers. It is evident from the data that the concentration of uracil was greater in the wax slice compared to the other three parts (*p* < 0.05). There was no significant difference among the other three groups (*p >* 0.05). Additionally, the inosine content in the wax slice was strongly significantly greater than other three parts (*p* < 0.01), while both the powder slice and gauze slice exhibited significantly higher levels of inosine than the bone slice (*p* < 0.01). Moreover, the hypoxanthine content was significantly elevated in both the wax slice and powder slice in comparison to the gauze slice and bone slice (*p* < 0.05).

### 3.4 Determination of amino acids contents

The Table 2 presents the amino acid compositions of different parts of deer antlers. It reveals a gradual decline in the levels of 17 amino acids from the upper to the basal section. Notably, the wax slice exhibited significantly higher concentrations of Asp, Met, and Phe compared to the powder slice, gauze slice, and bone slice (*p* < 0.05). Furthermore, the powder slice and gauze slice displayed significantly higher levels of these amino acids than the bone slice (*p* < 0.05). Additionally, the Thr content was significantly elevated in both the wax slice and powder slice compared to the gauze slice and bone slice (*p* < 0.05).

**TABLE 2:** Content of amino acid in different parts of deer antler.

Item	Wax slice	Powder slice	Gauze slice	Bone slice
Asp	56.35 ± 2.74^a^	53.77 ± 1.89^b^	53.27 ± 1.20^b^	52.74 ± 1.54^c^
Thr	21.39 ± 2.11^a^	21.38 ± 1.45^a^	19.58 ± 1.24^b^	18.75 ± 1.81^b^
Ser	47.06 ± 3.14	47.05 ± 1.25	47.00 ± 1.07	46.97 ± 1.54
Glu	76.65 ± 2.19^A^	70.56 ± 2.74^B^	64.25 ± 4.32^C^	60.57 ± 3.18^D^
Gly	137.89 ± 9.24^A^	130.52 ± 5.21^B^	124.28 ± 5.27^C^	123.14 ± 3.33^C^
Ala	53.65 ± 2.78	53.44 ± 1.20	53.01 ± 1.44	52.97 ± 1.01
Cys	46.48 ± 3.24	46.12 ± 2.19	46.10 ± 1.87	46.02 ± 3.74
Val	21.99 ± 1.20	21.39 ± 1.98	21.02 ± 1.57	20.99 ± 2.10
Met	8.85 ± 0.87^a^	8.21 ± 1.24^b^	8.19 ± 1.27^b^	7.64 ± 1.33^c^
Lle	43.61 ± 4.65	43.57 ± 2.58	43.21 ± 2.11	43.01 ± 1.98
Leu	70.59 ± 7.25^a^	65.24 ± 5.21^b^	61.24 ± 3.57^c^	59.24 ± 1.68^c^
Tyr	20.13 ± 2.14	20.11 ± 1.24	20.01 ± 1.08	19.95 ± 1.57
Phe	21.2 ± 1.20^a^	19.25 ± 2.45^b^	19.04 ± 1.56^b^	17.24 ± 2.00^c^
Lys	52.01 ± 3.67^a^	50.11 ± 2.65^b^	48.27 ± 3.14^c^	47.52 ± 2.14^c^
His	41.32 ± 2.24^A^	35.74 ± 3.51^B^	29.57 ± 1.34^C^	22.10 ± 1.88^D^
Arg	34.66 ± 2.17^a^	31.25 ± 1.25^b^	31.00 ± 1.17^b^	30.85 ± 1.55^b^
Pro	48.48 ± 3.25^A^	39.21 ± 1.27^B^	39.07 ± 2.47^B^	38.99 ± 2.40^B^

Note: Means with different lowercase letters within a row differ significantly (*p* < 0.05). Means with different uppercase letters differ extremely significantly (*p* < 0.01).

The levels of Glu and His in different parts were strongly significantly differences (*p* < 0.01). Moreover, the Gly content was notably higher in the wax slice than in the remaining three parts (*p* < 0.01), and significantly higher in the powder slice than in the gauze slice and bone slice (*p* < 0.01). Likewise, both Leu and Lys contents were significantly higher (*p* < 0.05) in the wax slice than in the other three parts, and also significantly higher (*p* < 0.05) in the powder slice than in the gauze slice and bone slice. The Arg content was significantly elevated (*p* < 0.05) in the wax slice compared to the other three parts. Lastly, the Pro content in the wax slice was highly significantly higher than the remaining three parts (*p* < 0.01).

### 3.5 Analysis of mineral element composition

Table 3 presents the mineral element composition in different parts of deer antlers. The data reveals that the potassium (K) content in the wax slice and powder slice was significantly higher than that in the gauze slice and bone slice (*p* < 0.01). Furthermore, the gauze slice exhibited a significantly higher K content than the bone slice (*p* < 0.01), and the wax slice group had a notably higher K content than that in the powder slice (*p* < 0.05). The sodium (Na) and magnesium (Mg) contents across different antler parts demonstrated significant variations (*p* < 0.05), with Na content declining gradually from the upper to the basal section of deer antlers, while Mg content exhibited an opposite trend. Additionally, the zinc (Zn) content in the wax slice, powder slice and gauze slice was significantly higher than the bone slice (*p* < 0.05). Conversely, the iron (Fe) content was markedly lower in the wax slice compared to the other three parts (*p* < 0.01), and it was notably lower in the powder slice and gauze slice than in the bone slice (*p* < 0.01), with a significant difference also observed between the powder slice and gauze slice (*p* < 0.05).

**TABLE 3:** Content of mineral elements in different parts of deer antler.

Item	Wax slice	Powder slice	Gauze slice	Bone slice
K	7.62 ± 0.119^aA^	6.48 ± 0.163^bA^	5.00 ± 0.130^B^	3.17 ± 0.121^C^
Na	8.66 ± 0.115^a^	8.15 ± 0.140^b^	7.84 ± 0.132^c^	7.23 ± 0.140^d^
Mg	3.54 ± 0.11^d^	3.77 ± 0.56^c^	4.06 ± 0.38^b^	4.29 ± 0.19^a^
Zn	0.074 ± 0.001^a^	0.071 ± 0.003^a^	0.068 ± 0.002^a^	0.064 ± 0.002^b^
Fe	0.087 ± 0.008^C^	0.113 ± 0.008^bB^	0.128 ± 0.007^aB^	0.156 ± 0.011^A^

Note: Means with different lowercase letters within a row differ significantly (*p* < 0.05). Means with different uppercase letters differ extremely significantly (*p* < 0.01).

### 3.6 Effect of different parts of deer antler on immunological activity in mice

**FIGURE 5:**
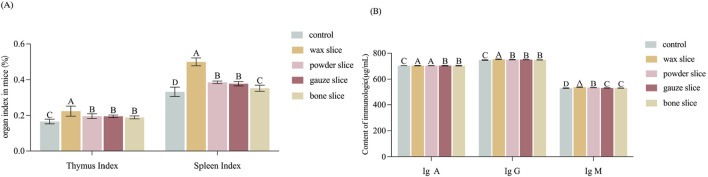
Effect of different parts of deer antler on immunological activity in mice **(A)** Effect of different parts of deer antler on organ index in mice (n = 20). **(B)** Effect of in different parts of deer antler slice on immunologic (n = 20) Note: Means with different uppercase letters differ extremely significantly (*p* < 0.01). Control: The mice in control group were administered 0.3 mL of saline solution per day, continuously for 14 days from the first day. Wax slice: The mice in wax slice group were administered 0.3 mL of wax slice enzymatic hydrolysate per day, continuously for 14 days from the first day. Powder slice: The mice in powder slice group were administered 0.3 mL of powder slice enzymatic hydrolysate per day, continuously for 14 days from the first day. Gauze slice: The mice in gauze slice group were administered 0.3 mL of gauze slice enzymatic hydrolysate per day, continuously for 14 days from the first day. Bone slice: The mice in bone slice group were administered 0.3 mL of bone slice enzymatic hydrolysate per day, continuously for 14 days from the first day (The following figure is the same.).

The index of thymus and spleen serve as indicative measures of the organism’s immune function. Figure 5A illustrates that the organ index of mice administered with the enzymatic hydrolysate of different parts of deer antler were all significantly higher than those of the control group (*p* < 0.01). Notably, the thymus index exhibited a gradual decrease from the upper to the basal sections and was significantly higher in the wax slice group compared to the powder slice, gauze slice and bone slice groups (*p* < 0.01). Conversely, there was no significant difference observed between the powder slice, gauze slice, and bone slice groups (*p* > 0.05). Similarly, the spleen index also displayed a gradual decrease from the upper to the basal sections and was significantly higher in the wax slice group compared to the powder slice, gauze slice, and bone slice groups (*p* < 0.01). Moreover, there was no significant difference observed between the powder slice and gauze slice groups (*p* > 0.05); however, both were significantly higher than the bone slice group (*p* < 0.01).

The impact of deer antler enzyme digest on the immunological activity of mice was assessed by measuring the secretion of cytokines IgA, IgG, and IgM. As depicted in Figure 5B, the serum levels of IgA exhibited a gradual decrease from the upper to the basal sections. The levels of IgA, IgG, and IgM in the experimental group were all strongly significantly than those in the control group (*p* < 0.01). Furthermore, the IgG levels in the wax slice group was strongly significantly than those in the control group and the other experimental groups (*p* < 0.01). Similarly, the IgM levels in the wax slice group was strongly significantly than those in the powder slice, gauze slice and bone slice groups (*p* < 0.01). Additionally, The serum IgA content in the wax slice and powder slice was significantly greater than that in the gauze slice and bone slice group (*p* < 0.01), while there was no significant difference between the gauze slice group and the bone slice group (*p* > 0.05).

### 3.7 Effect of different parts of deer antler on anti-fatigue in mice

**FIGURE 6:**
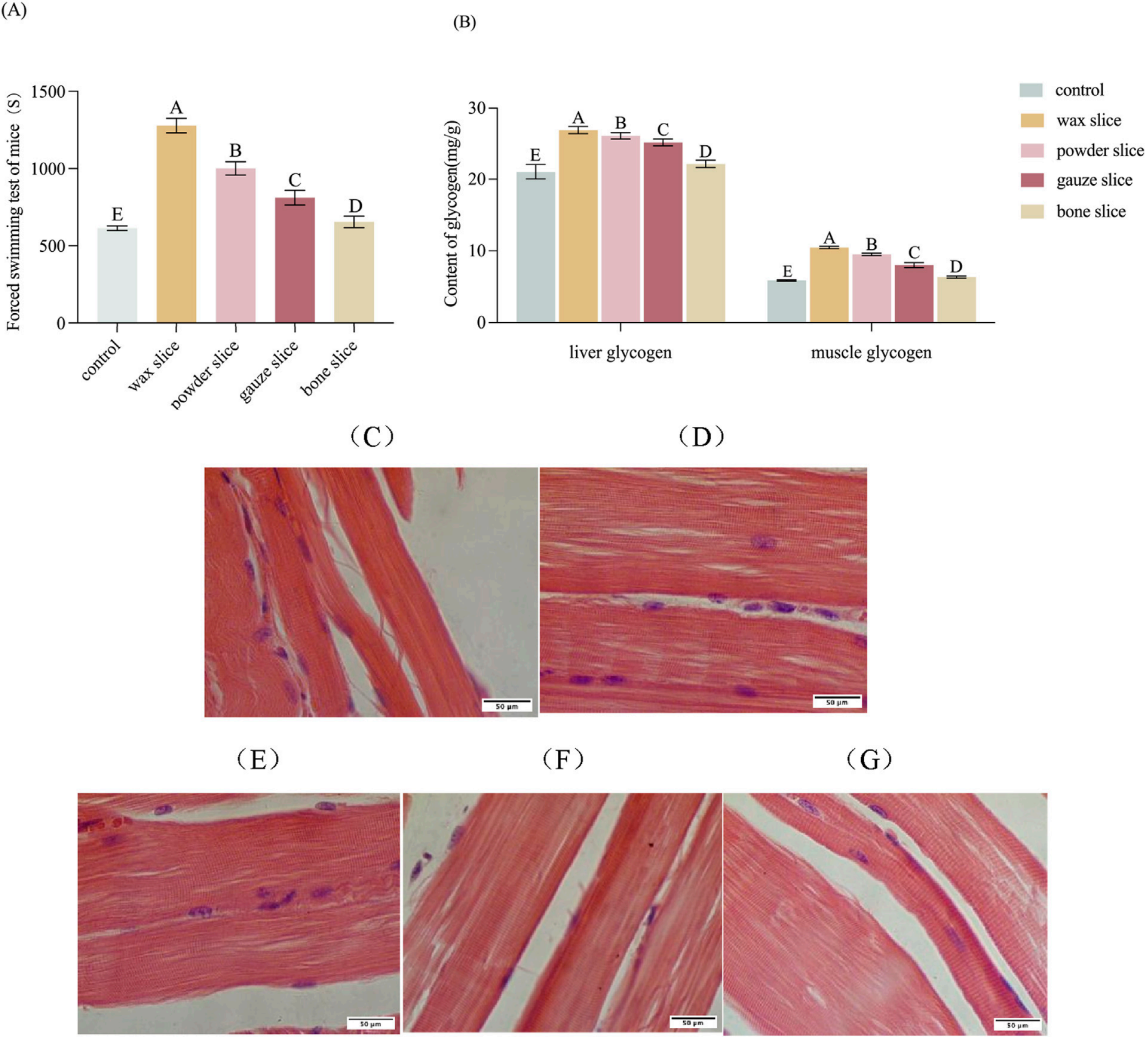
Effect of different parts of deer antler on anti-fatigue in mice. **(A)** Effect of different parts of deer antler on the swimming time of loading mice (n = 20). **(B)** Effect of different parts of deer antler on glycogen content of mice (n = 20). **(C)** Hematoxylin and Eosin (H&E)-stained of muscle sections after saline solution treatment (n = 20). **(D)** Hematoxylin and Eosin (H&E)-stained of muscle sections after wax slice enzymatic hydrolysate treatment (n = 20). **(E)** Hematoxylin and Eosin (H&E)-stained of muscle sections after powder slice enzymatic hydrolysate treatment (n = 20). **(F)** Hematoxylin and Eosin (H&E)-stained of muscle sections after gauze slice enzymatic hydrolysate treatment (n = 20). **(G)** Hematoxylin and Eosin (H&E)-stained of muscle sections after bone slice enzymatic hydrolysate treatment (n = 20). Note: Means for different lowercase letters were significantly different (*p* < 0.05). Means with different uppercase letters differ extremely significantly (*p* < 0.01).

The impact of different parts of deer antler on the forced swimming endurance of mice is displayed in Figure 6A. The forced swimming duration of mice in the experimental groups was significantly longer than that of the control group (*p* < 0.01). There was a significant difference observed among deer antler groups (*p* < 0.01), with the endurance ranking in the following order: wax slice > powder slice > gauze slice > bone slice.

The impact of different parts of deer antler on the glycogen content of mice is presented in Figure 6B. The hepatic and muscular glycogen levels in the experimental groups were significantly elevated compared to those in the control group (*p* < 0.01). Furthermore, there were significant differences in the levels of hepatic and muscular glycogen among the deer antler groups (*p* < 0.01), with the following ranking from highest to lowest: wax slice > powder slice > gauze slice > bone slice.

To evaluate the impact of enzymatic hydrolysate administration of different parts of deer antler on the morphology of skeletal muscle in mice, the histology of skeletal muscle was assessed through H&E staining (Figures 6C–G). The muscle fibres and transverse striations appeared more relaxed in the control group and tighter in the test group. The wax slice group displayed the most favorable skeletal muscle condition in mice, characterized by distinct transverse striations and taut muscle fibre. There was no significant difference between the powder slice group and the gauze slice group, while the transverse striations of the bone slice group were apparent but less taut.

### 3.8 Correlation between chemical composition and immune-enhancing effects

**FIGURE 7:**
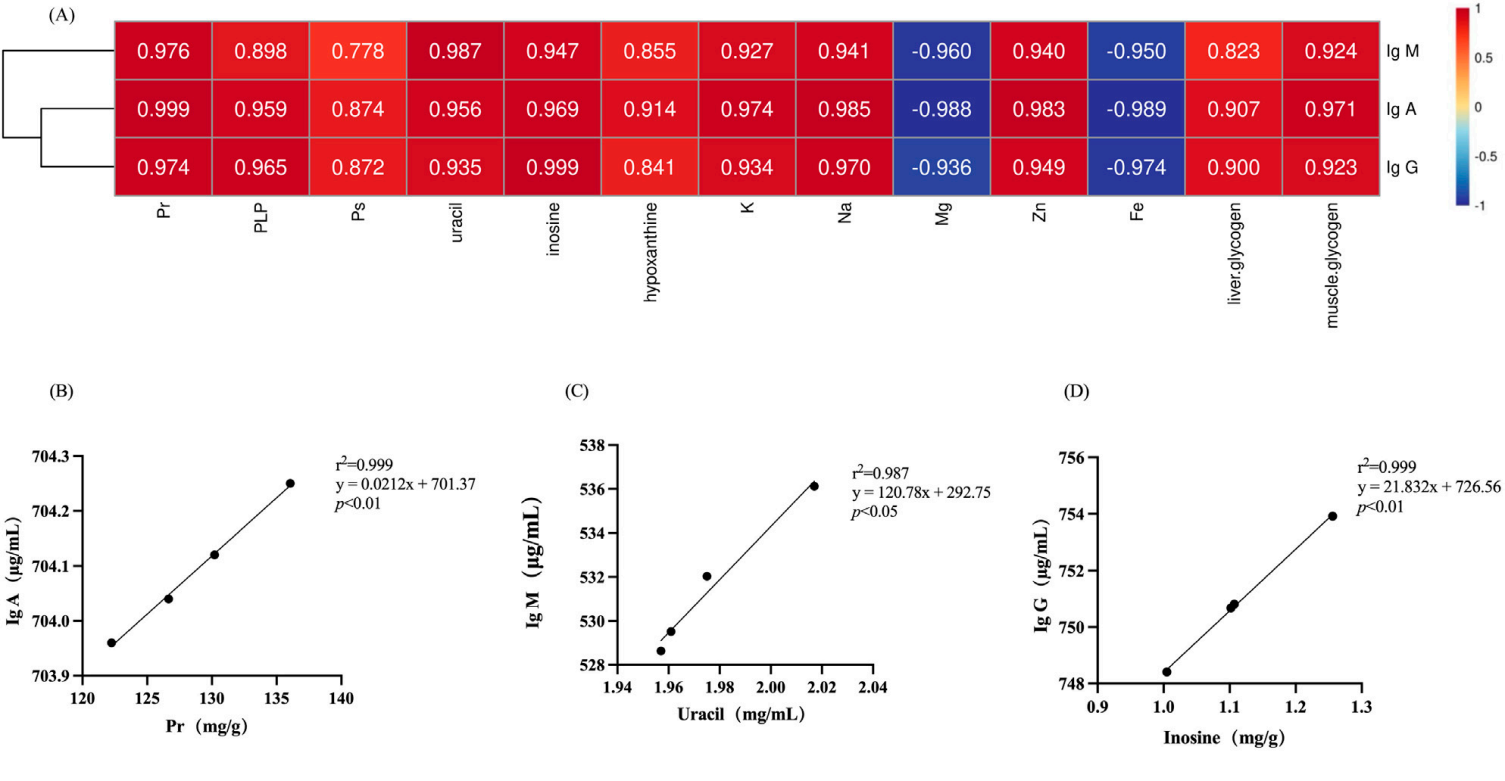
Correlation of chemical compositions in deer antlers with immunological indicators. **(A)** Heatmap of the correlation between chemical compositions in deer antler and immune indicators. **(B)** Simple linear regression analysis of protein versus IgA. **(C)** Simple linear regression analysis of uracil versus IgM. **(D)** Simple linear regression analysis of inosine versus IgG. Note: Different lower case letters indicate significant differences (*p* < 0.05) and different upper case letters indicate highly significant differences (*p* < 0.01). (Pr, protein; PLP, phospholipid; PS, polysaccharide; same as follow).

Correlation analysis was employed to ascertain the correlation between various types of compositions in deer antler and immune-enhancing effect. As depicted in Figure 7, among the organic and inorganic compositions of deer antler, the highest degree of correlation with the content of IgA, IgM, and IgG was found to be protein (r^2^ = 0.999), uracil (r^2^ = 0.987), and inosine (r^2^ = 0.999), respectively.

### 3.9 Correlation between chemical composition and anti-fatigue effects

**FIGURE 8:**
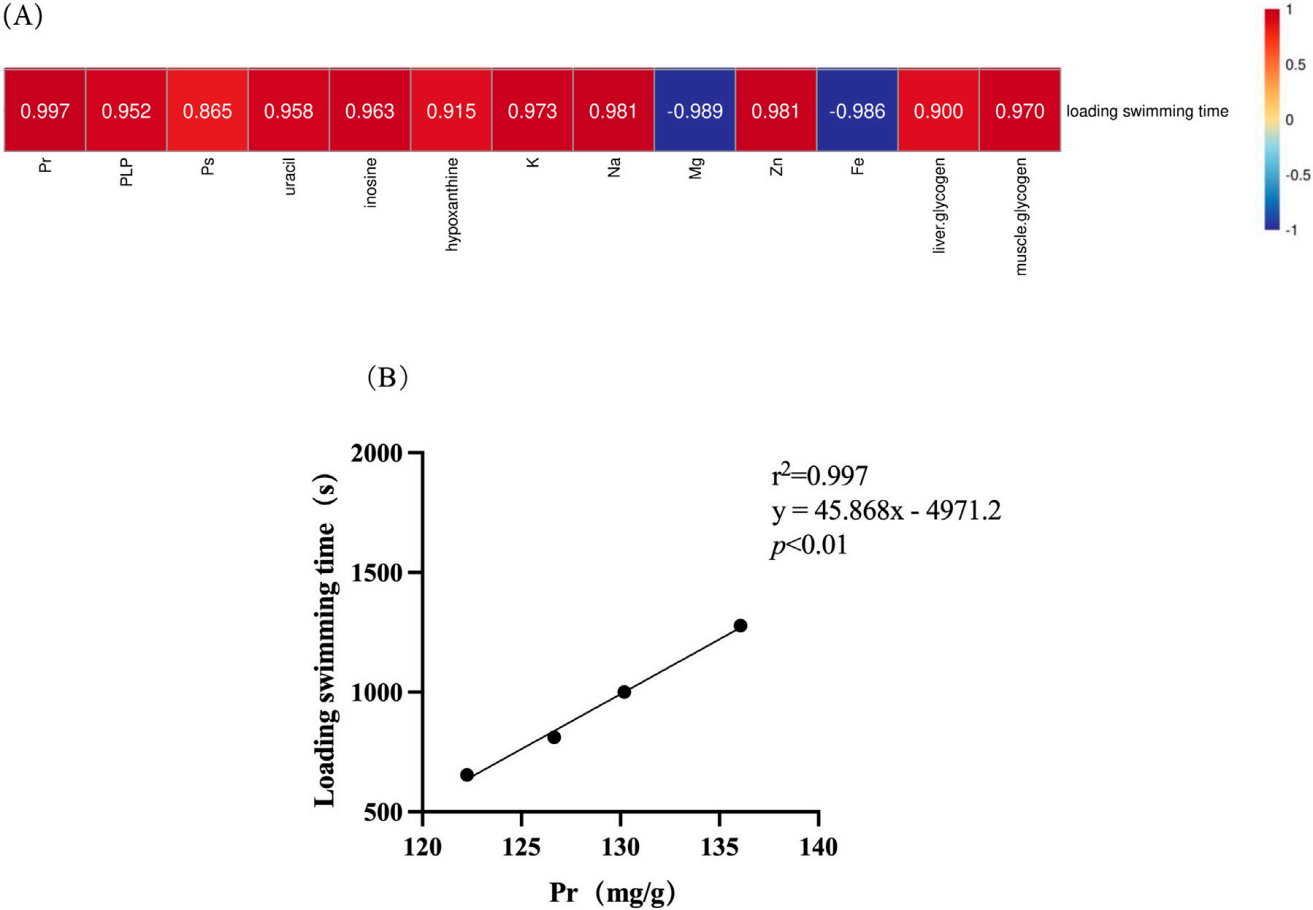
Correlation of chemical composition in deer antlers with endurance swimming duration. **(A)** Heatmap of the correlation between chemical compositions in deer antler and endurance swimming duration. **(B)** Simple linear regression analysis of protein levels versus endurance swimming duration. Note: Different upper case letters indicate highly significant differences (*p* < 0.01).

Figure 8 illustrates the correlation between chemical composition and the anti-fatigue properties. It is evident that proteins (r^2^ = 0.997) appear to exert a significant influence on the anti-fatigue effect, while polysaccharides (r^2^ = 0.865) demonstrate the least relevance.

## 4 Discussion

Deer antler, a traditional animal medicine, possesses diverse medicinal properties. The immunomodulatory and anti-fatigue properties of antler have been unequivocally demonstrated; however, limited research has investigated the effects of different parts of antler when administered separately. In consideration of the intricate composition of marker substances in animal medicines, this study initially examined the protein, amino acid, polysaccharide, phospholipid contents in wax, powder, gauze and bone slices respectively. Subsequently, the effects of enzymatic hydrolysate derived from different parts of antlers on immune enhancement and anti-fatigue properties in mice were investigated. Finally, the composition and efficacy were examined through correlation analysis to provide a comprehensive evaluation in accordance with academic standards.

The assessment of visual qualities in deer antler slices typically relies on subjective evaluations based on sensory perception and experience, lacking quantitative criteria and being susceptible to individual biases. The inconsistent quality of deer antler slices in the market poses a challenge in distinguishing genuine products from counterfeit ones, highlighting the limitations associated with relying solely on subjective assessment. Therefore, this study conducted a comparative analysis of the morphological characteristics observed in different sections of deer antler slices, aiming to provide a more comprehensive and visually accessible approach for identifying specific parts of deer antlers. Consequently, this research facilitates the precise application and utilization of distinct components within deer antlers.

The utilization of deer antler as a raw material for the enzymatic hydrolysis-based production of deer antler polypeptide has been predominant in recent years. The deer antlers were partitioned into four segments, namely, wax slice, powder slice, gauze slice, and bone slice, and subsequently subjected to enzymatic hydrolysis to obtain the deer antler enzymatic hydrolysate. The potency of the pharmacological effects of deer antler is directly influenced by the concentration of marker substances it contains.

The pricing of various parts of deer antler in the market is, to a certain extent, determined by this factor. Therefore, it is imperative to ascertain the content of marker substances in each component of deer antler. The protein, phospholipid, and polysaccharide contents in the enzymatic hydrolysate of different deer antler parts were subsequently quantified. The results revealed a declining trend in the protein, phospholipid, and polysaccharide contents across the wax slice to the bone slice of deer antler enzymatic hydrolysate.

Deer antlers are abundant in proteins, amino acids, and polypeptides, which serve as essential biomarkers in the field of deer antler research (Jeon et al., 2008). The results of a comprehensive analysis revealed that proteins exhibited the strongest correlation with IgA levels and endurance swimming time, highlighting the pivotal role of proteins in mediating the diverse pharmacological effects of deer antler. Kim and colleagues (Kim et al., 2003) identified a total of 16 amino acids in deer antler through hydrolysis, with Asp, Glu, Pro, Gly, and Arg collectively accounting for approximately 32.5%–37.2% of the overall amino acid composition. In the present study, a total of 17 amino acids were identified, and there were no significant disparities observed in the overall amino acid composition among different parts of deer antlers. The predominant amino acids, namely, Aspartic acid (Asp), Glutamic acid (Glu), Leucine (Leu), Glycine (Gly), and Alanine (Ala), collectively accounted for approximately 50% of the total amino acid composition. Additionally, the relative proportion of individual amino acids displayed a gradual decrease from the upper to basal sections, despite constituting a smaller fraction within deer antler. The evidence presented fully substantiates the superior nutritional value of wax slices.

The amino acids play a pivotal role as the fundamental building blocks of polypeptides. The amino acid content and peptide content in each part exhibit a consistent trend. Zha E and colleagues (Zha et al., 2013) reported the isolation of a 3.2 kDa polypeptide, nVAP32, from deer antler, which exhibited a dose-dependent modulation of immune activity in mice. Organisms are comprised of a diverse array of substances, with phospholipids emerging as a prominent class of lipid compounds characterized by the presence of phosphoric acid, playing a crucial role in the constitution of biological membranes. The results of this investigation revealed a declining trend in phospholipid levels across different sections of deer antler, from the wax slice to the bone slice. These findings suggest that the wax slice may possess superior wound-healing properties compared to the bone slice. The wax slice is situated atop the deer antler, which is recognized as the tissue exhibiting the highest growth rate and lowest cancer incidence (Li et al., 2023). This can be attributed to the continuous phosphorylation of stromal cells during the antler’s growth process. Hence, the pivotal role of protein and phosphate in the pharmacological effects of deer antler cannot be overstated.

In recent years, the continuous advancement of research methodologies has propelled peptides and polysaccharides to the forefront of animal and plant medicine pharmacology investigations, garnering significant attention from both domestic and international scholars. Polysaccharides, as a class of biologically active macromolecules devoid of toxicity, have gained significant attention in recent years for their potential in the development of health products. Multiple studies have demonstrated the therapeutic potential of deer antler polysaccharides in augmenting human immune function (Li et al., 2002). Although the correlation results in this experiment indicate a modest association between polysaccharides and immune indicators, the observed trends in polysaccharide content and immune indicators across the four experimental sections consistently demonstrated a gradual decrease from the upper to basal regions. Therefore, it is widely acknowledged that polysaccharides can serve as suitable indicators for assessing the quality of various components of deer antler.

The minerals present in deer antler can be categorized into major elements and trace elements, both of which play crucial roles in facilitating the medicinal properties of deer antler. In this experiment, the concentrations of three inorganic substances, namely, potassium (K), sodium (Na), and magnesium (Mg), were analyzed across distinct sections of deer antlers. Potassium (K) and sodium (Na) play pivotal roles in maintaining the acid-base balance and osmotic pressure within the animal organism, being actively transported via the Na-K pump. The concentrations of these ions are higher both intracellularly and extracellularly, particularly in regions of active substance exchange, such as the rapidly growing tissues found at the apex of deer antlers with a wax slice. Consequently, the uppermost segment of deer antlers demonstrates the highest concentrations of Na and K, exhibiting a gradual decline in levels towards the basal section. Previous studies have demonstrated that sodium possesses the ability to modulate other subsets of T-lymphocytes associated with autoimmune diseases and allergies. CD8-positive T-lymphocytes play a pivotal role in the adaptive immune system, and it has been demonstrated that sodium ions augment the activity of the sodium-potassium pump in T-lymphocyte membranes, thereby bolstering the immune response of CD8 T-cells (Soll et al., 2024). Hence, the presence of Na and K exerts a direct influence on augmenting immune functionality. The populations of osteoblasts and osteoclasts gradually emerge in the central and basal regions of deer antler, with calcium (Ca), phosphorus (P), and magnesium (Mg) serving as the primary constituents of the bone slice (Zuo and Yu, 2013). The levels of these three elements will progressively increase during the process of ossification. Despite their minute presence within the body, trace elements play an indispensable role in the development of living organisms.

The pharmacological effects of deer antlers are diverse, with the enhancement of the body’s immune activity being one of its primary impacts. The thymus and spleen function as crucial immune organs, with their organ indices serving as direct indicators of the body’s level of immune functionality (Zhu et al., 2019). The impact of drugs on the spleen and thymus indices can serve as an initial indicator for investigating immunopharmacological mechanisms in animal models. A higher organ index substantiates a stronger immune response, and in conjunction with the findings of this study, it is evident that wax slices exhibit the most pronounced effect, followed by powdered slices, gauze slices, and bone slices in descending order. All these treatments significantly surpass the control group. The immunoglobulin is a type of immunoreactive substance predominantly found in serum. The immunoglobulin content level of the organism is positively associated with the efficacy of diverse biological functions, including antiviral and antibacterial activities within the animal body (Li, 2018).

In our investigation, we observed a significant increase in the levels of IgG and IgM in the wax slice group compared to all other experimental groups. Moreover, the concentration of IgA in both the wax slice and powder slice groups exhibited a significantly higher level compared to that observed in the gauze slice and bone slice groups. The results strongly support the superior efficacy of wax slice of deer antler in boosting the immune system.

Additionally, the continuous administration of enzymatically hydrolyzed deer antler extract to mice resulted in a significant augmentation in weight-bearing swimming duration, along with heightened levels of liver glycogen and muscle glycogen content within the murine hindlimb muscles. The research findings have indicated that deer antler peptides demonstrate anti-fatigue effects through the enhancement of skeletal muscle troponin-related mRNA expression, upregulation of muscle contraction-related genes to augment muscle strength, and improvement in various fatigue indicators such as blood glucose, blood urea, and lactic acid levels (Chen et al., 2014).

Nucleosides, although present in relatively low quantities, constitute a class of biomarkers in deer antler with multifaceted functions encompassing immunomodulation, anti-cancer properties and hepatoprotection. The biological functions of deer antler are intricately linked. Revised sentence: Consequently, the nucleoside content can serve as an indicator for assessing the quality of deer antler (Zhao et al., 2020). The levels of three nucleosides, namely, uracil, inosine, and hypoxanthine, were determined in this study. Remarkably, the concentrations obtained were consistent with those reported by Zhou and Li (2009). The correlation analysis revealed a robust association between uracil, inosine, and the *in vivo* levels of IgM and IgG. This implies an inherent connection between the immunomodulatory pharmacological effects of deer antler and its nucleoside composition, suggesting a potential interdependence. Some studies have demonstrated that deer antler possesses the ability to inhibit triple-negative breast cancer through certain pathways in the immune system, indicating that specific marker substances within deer antler not only regulate immune function but also modulate cancer response via immunomodulation (Li et al., 2022). Additionally, Zhao et al. (2020) identified a total of eight nucleosides in deer antler and conducted a weight-bearing swimming experiment in mice to evaluate the anti-fatigue activity of different components of deer antler. Their findings revealed a gradual increase in the swimming duration of mice, highlighting the potential fatigue-reducing properties of deer antler.

In general, clinical trials have consistently demonstrated the profound impact of deer antler on augmenting immune response and mitigating fatigue, establishing it as a highly effective dietary supplement. The identification of the specific marker substances responsible for these effects necessitates further investigation. Furthermore, our study exclusively employed the enzymatic hydrolysate of deer antler for experimentation. Relevant future research endeavors may encompass the investigation of alternative formulations of deer antler to augment our comprehension of its pharmacological properties.

## 5 Conclusion

The results of this study indicate that the concentration of marker substances in wax slices of deer antler gradually decreases from the upper to the basal sections, with the highest concentration observed in the former. The protein exhibited the highest correlation coefficients with Ig A and anti-fatigue, uracil showed the strongest association with Ig M, and inosine demonstrated a significant correlation with Ig G. The data presented in this study provides evidence for the immunomodulatory and anti-fatigue properties of deer antler, supporting its potential as a valuable therapeutic agent.

## Data Availability

The original contributions presented in the study are included in the article/supplementary material, further inquiries can be directed to the corresponding author.
